# Comparative energy demand and carbon footprint analysis of textile waste management systems in Australia

**DOI:** 10.1007/s11356-025-36200-1

**Published:** 2025-03-14

**Authors:** Mahbuba Imroz Khan, Md Tasbirul Islam, Lijing Wang, Rajiv Padhye

**Affiliations:** 1 School of Fashion and Textiles, RMIT University, Melbourne, Australia; 2 Interdisciplinary Research Center for Sustainable Energy Systems (IRC-SES), King Fahd University of Petroleum & Minerals, Dhahran, 31261 Saudi Arabia

**Keywords:** Recycling, Incineration, Cotton waste recycling, Energy demand, CO_2_ emission, Textile waste management

## Abstract

In modern societies, especially in developed countries, textile waste management has become a critical issue due to overconsumption and throw-away culture. This case study aims to develop a comprehensive quantitative energy demand and carbon footprint analysis model using CCalC2 software to identify the most sustainable end-of-life management practices for the textile waste in Australia, the second-largest consumers of textile products. Results of this study showed that chemical recycling is the best option from the standpoint of saving pulp production energy and recycled pulp material, while it is a carbon-intensive process (6401 kg CO_2_eq/1000 kg). The mechanical recycling process is estimated to emit around 5368 kg CO_2_eq/1000 kg — a low CO_2_ emitting process. Surprisingly, CO_2_ emissions from incineration (e.g., 5897 kg CO_2_eq/1000 kg) showed a similar trend of mechanical recycling, and the option of incineration could only be pursued if (thermal) energy recovery and electricity production were considered. This study highlights the requirement for sustainable textile waste management practices and provides valuable insights for policymakers and industry stakeholders for future policy planning around low carbon-intensive technology selection with improved (secondary) material recovery.

## Introduction

The end-of-life (EoL) stage of textile waste management has become a critical challenge due to its substantial environmental impact. Global greenhouse gas emissions and environmental pollution are significantly contributed by the waste stream, which is generally disposed of in landfills and incinerators. In response to this impact, the textile industry is exploring management alternatives for EoL treatments, such as large-scale separation and recycling, which is vital for CO_2_ equivalent (CO_2_eq) emission reduction and environmental impact (Zhou et al. 2022).

**Fig. 1:**
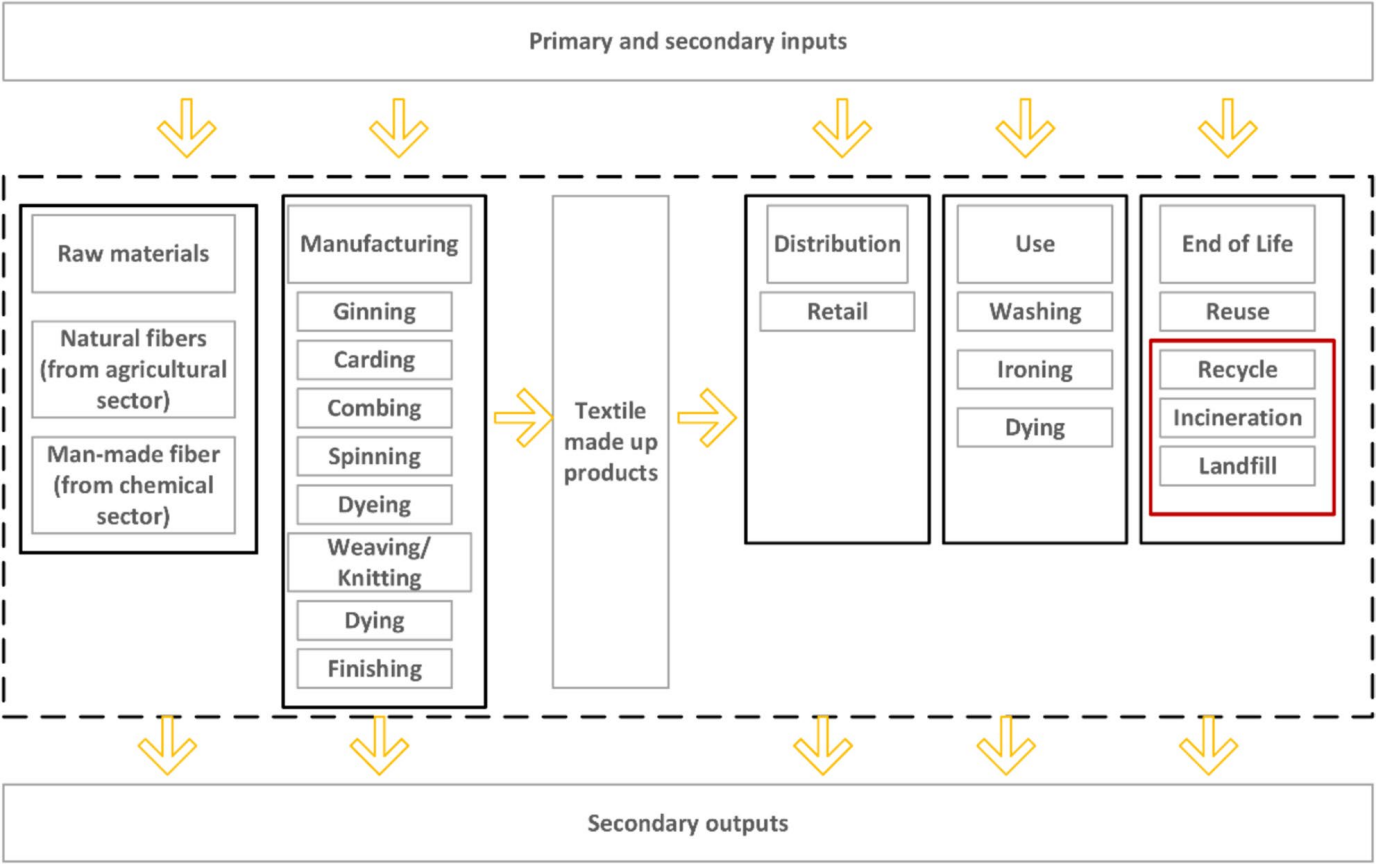
Typical textile process flow and focus of this study (adapted and redrawn from (Amicarelli et al. 2022))

Recently, environmental burdens associated with the textile industry have been researched considerably. Liu et al. (2022) and Yoro and Daramola (2020) concluded that while the production stage is known to be the greenhouse gas (GHG) emission hotspot, managing textile waste at EoL is equally essential in order to achieve carbon footprint (CF) reduction and address resource depletion. Figure 1 shows lifecycle stages of textile products. The EoL process as indicated in the box outlined in red is the focus of this investigation.

The annual textile waste generation globally recorded around 92 million tons (Mt), among which a significant portion of the waste ended up in landfills or being incinerated (Ruiz 2023). This unsustainable EoL management practice calls for a shift towards a more environmentally sustainable practice, such as recycling and reuse.

Each year, Australian residents collectively discard approximately 23 kg of clothing per individual, culminating in a substantial 93% of the garments being relegated to textile waste. Predominantly, this waste is directed to landfill sites in Australia, a practice that notably exacerbates global warming (Moazzem et al. 2021a). Among various waste categories, textiles manifest the lowest recovery rate, with a substantial 87.5% of these materials eluding recycling processes and ultimately contributing to landfill accumulation (Khan et al. 2023). From the Australian context, textile waste and its environmental impact, especially at the EoL stage and various alternative pathways, are relatively underexplored. Environmental impacts of pre-treatment methods and the comparison of recycling and landfilling processes are some of the initial studies conducted by Rosson and Byrne (2020) and Moazzem et al. (2021b), respectively.

In order to justify the novelty of this work, a literature review needs to be performed. Textile waste management in Australia was first reviewed (Khan et al. 2023). A further comprehensive literature review was conducted on the carbon footprint of textile waste. To conduct this review, the Web of Science Core Collection database was searched using the keyword “textile waste,” yielding 2701 articles. This search was subsequently refined with the term “carbon footprint,” narrowing the results to 20 relevant articles, which were analyzed and summarized in Table 1.

**Table 1:** Summary of the literature

Reference	Country	Major findings	Material/waste considered	Methodology	Software	Waste management alternatives
Pérez et al. (2022)	Chile	Recycling reduces carbon emissions by 5778 kg CO₂ per ton, significantly lowering the environmental impact compared to landfilling	Textile waste	Life cycle assessment (LCA)	The ReCiPe 2016 midpoint (H) methodology and the database Ecoinvent 3	Recycling process
Morell-Delgado et al. (2024)	Spain (Catalonia)	In Catalonia, 90% of municipal textile waste is landfilled or incinerated, with only 10% properly collected for reuse or recycling	Textile waste	A combination of citizen surveying and environmental quantitative tools, such as material flow analysis and life cycle assessment	SimaPro software, IBM SPSS statistic software	Textile waste disposal practices
Zhang et al. (2024)	China	MXene-coated recycled cellulose fibers enhance the value of textile waste by enabling its use in fireproof and EMI-shielding composites for high-performance applications	Wasted cellulose fiber	The opening carding-needling punch technique		Recycling of waste cellulose fiber
Yousef et al. (2019)	Lithuania	Pyrolysis, enhanced by dye metals acting as self-catalysts, offers a sustainable alternative to landfilling for textile waste by improving conversion efficiency and enabling bioenergy recovery	Waste jeans	Pyrolysis		Recycling
Xie et al. (2024)	China	Catalytic pyrolysis as a promising method for upcycling textile waste into valuable fuels and chemicals, reducing environmental impact while creating economic opportunities	Textile waste	Literature review, LCA		Recycling
Vera et al. (2024)	USA	A dual-process system that converts cotton textiles into glucose and recovers polyester fibers boosts textile circularity and sustainability while improving the financial viability of textile waste valorization	Cotton waste and cotton polyester blend waste	Techno economic analysis and LCA	OpenLCA and Ecoinvent 3.8	Recycling
Wu et al. (2015)	China	Jeans using mixed cotton fiber reduced water footprint by 26.18% compared to 100% virgin cotton, mainly due to less water usage in fiber production and dyeing, with a smaller reduction in carbon footprint due to dyeing omission	Jeans made of mixed cotton fiber and virgin cotton fiber	Water footprint and carbon footprint		Recycling
Rosson and Byrne (2020)	Australia	Acid pre-treatment in cotton recycling is more environmentally friendly than alkali pre-treatment, due to lower energy use and emissions	Waste cotton	LCA	SimaPro 9.0.0.48 with Ecoinvent database	Chemical recycling
Berger and Pfeifer (2024)	Austria	Blended PET compositions, like 40% bio-PET and 60% recycled PET, offer a sustainable yet cost-effective solution, though bio-PET's financial viability depends on government subsidies or process optimization	Polyester textile waste	Techno-Economic Analysis and LCA	SuperPro Designer and Microsoft Excel, Monte Carlo Simulation Refinery Tool	Recycling
Muthu et al. (2012)	Hong Kong	Recycling pre-consumer textile waste is more efficient than post-consumer recycling due to challenges in fiber sorting and processing, and it calls for government incentives, extended producer responsibility, and greater adoption of eco-design principles	Cotton and polyester textile waste	LCA	SIMAPRO	Recycling of pre- and post-consumer textile waste
(Islam et al. 2022)	Australia	Examines the barriers and enablers for recycling-focused circular business models across various industries, including textiles, emphasizing the role of digital tracking technologies (such as AI, blockchain, and RFID) and extended producer responsibility in facilitating their implementation	Textile waste	Literature review	Content analysis, text mining using NVivo software, and framework matrix for data structuring	
Lin et al. (2024)	Taiwan	Explores the use of electrodialysis and bipolar membrane electrodialysis (BMED) to recover water and chemicals in PET recycling, finding that BMED can reduce the water footprint of PET recycling by tenfold while also enabling chemical recovery for circular economy applications		Carbon footprint (CF) and water footprint (WF)		
Zamani et al. (2015)	Sweden	Material reuse and fiber separation can reduce emissions by up to 10 tons of CO₂ per ton of textile waste, emphasizing the need for policy support, industrial-scale optimization, and increased public participation in recycling initiatives	Textile waste (50% cotton and 50% polyester)	LCA		Remanufacturing, cellulose-polyester separation, chemical polyester recycling and incineration
Aneja et al. (2024)	USA	Hydrolytic depolymerization can effectively recover polyester monomers (TPA and EG) without hazardous chemicals, making it a promising chemical recycling method	Polyester textile waste	Kinetic models for hydrolytic depolymerization		Chemical recycling
Alves et al. (2024)	Portugal	Recycled textile fibers can be converted into non-woven structures for construction, automotive, and packaging applications, with the optimization of mechanical, chemical, and bio-based recycling technologies for high-value textile waste applications	Textile waste	Literature review		
Violano and Cannaviello (2023)	Italy	Recycled textile insulation has a lower carbon footprint than conventional insulation materials like polystyrene and mineral wool	Textile waste	Comparative life cycle assessment		Recycling
Di et al. (2023)	Sweden	High-performance insulation foams from upcycled Kevlar nanofibers and nanocellulose, offering superior moisture resistance and sustainability for potential applications in green building, aerospace, and thermal packaging	Kevlar waste	Foam fabrication process		Recycling
Öndoğan et al. (2022)	Turkey	Circular economy models and sustainable material sourcing are emerging as competitive advantages, yet fast fashion overproduction remains a critical challenge that necessitates regulatory enforcement and increased consumer engagement		Qualitative analysis		
Reif et al. (2016)	Czech Republic	Natural fiber-based insulation materials (straw, hemp, and cellulose) offer strong thermal and acoustic performance but require moisture-resistant coatings for long-term durability	Cellulosic fibers, straw fibers, hemp fibers	Thermal conductivity analysis, water vapor resistance factor measurement, dynamic stiffness testing, acoustic performance evaluation		

In general, most of the studies considered various types of textile waste such as jeans waste and polyester waste with some considering cotton textile waste either fully or as part of a mixed waste stream. However, none of these studies has been conducted within the Australian context. This study specifically utilizes the composition of apparel waste based on the Australian textile waste ratio as model inputs. While several authors have analyzed cotton textile waste, only Wu et al. (2015) and Zamani et al. (2015) have incorporated energy demand alongside carbon footprint analysis. Notably, although cotton waste has been considered within the textile waste fraction as functional unit, only Wu et al. (2015) have conducted a carbon footprint assessment specifically on cotton textile waste. Other studies primarily employed life cycle assessment, which is beyond the scope of this research. Wu et al. (2015) implemented an analytical approach rather than relying on specialized software, whereas this study utilizes the CCalC2 software developed by the University of Manchester.

The majority of prior studies have focused on recycling as a preferred waste management strategy over landfilling, with a few explicitly addressing chemical recycling, such as Rosson and Byrne (2020), Aneja et al. (2024), and Zamani et al. (2015). This indicates a lack of comprehensive evaluations of different management alternatives. In contrast, the present study provides a comparative carbon footprint analysis of mechanical recycling, chemical recycling, incineration, and landfilling (as the baseline scenario), an approach that has not been previously undertaken in this manner within academic research in Australia. Carbon footprint analysis was selected due to its relative simplicity and comprehensibility for both academic and industry professionals. While LCA offers a more holistic system-level evaluation, this study serves as a preliminary assessment focusing on cotton textile waste within Australia, addressing a gap in the academic literature.

Table 1 highlights that only two studies from Australia met the search criteria outlined earlier. Islam et al. (2022) reviewed textile waste from a circular business model perspective, whereas Rosson and Byrne (2020) conducted an LCA focusing solely on the pre-treatment process of cotton chemical recycling rather than a system-level assessment of EoL alternatives. Thus, it can conclusively justify that this research contributes novelty addressing key areas, including the geographical context (with details provided in the methodology section), a comparative assessment of waste management alternatives, and a simplified yet robust analytical approach through carbon footprint assessment and energy demand estimation. These aspects have not been extensively explored in Australia or elsewhere.

This study aims to fill in the gaps by a comprehensive and comparative analysis of the Australian context in terms of energy demand and carbon footprint of various alternative EoL scenarios using CCalC2 software. The scope of this research encompasses with the EoL processing alternatives and its carbon footprint, and potential material and energy recovery from the textile waste stream (i.e., the last stage of Fig. 1, including landfill as the baseline scenario). The focus of this study is deliberately confined to the management of downstream textile waste (i.e., specific focus was on cotton portion of the textile waste for mechanical and chemical recycling), thereby excluding any consideration of upstream processes.

This study impacts local environmental conditions by contextualizing the EoL practices. It also addresses the fiber composition of waste generally found in Australia for textile waste. This study not only bridges the gaps between international insights and Australian applications but also paves the way for future research in evaluating sustainable textile waste management practices in other regions with similar environmental and economic conditions. This research provides valuable insights for policymakers and industry stakeholders for future policy planning around low carbon-intensive technology selection with improved (secondary) material recovery.

The research paper has two key research questions:

1. What is the energy demand of the EoL management alternative for Australian textile waste (i.e., landfilling, incineration, and recycling process of cotton, including mechanical and chemical recycling)?
2. What is the comparative carbon footprint of the management alternatives?

## Material and methods

### Goal and scope

The goal of this study is to estimate energy demand and CF analysis of various EoL treatment scenarios of apparel waste, namely (1) scenario 0, 100% landfill (as baseline); (2) scenario 1, 100% incineration; (3) scenario 2, mechanical recycling; and (4) scenario 3, chemical recycling.

The types of fibers associated with the textile waste, which serve as inputs, are a context-specific parameter. As Moazzem et al. (2021b) pointed out, the composition of apparel waste and the recycling processes are the two key factors in understanding the overall environmental benefits. In the case of Australian textile waste, the unique composition of natural and synthetic fibers is 64.4% and 35.6%, respectively. This ratio has been used as model inputs for the scenarios, making this study particularly relevant to the Australian context.

Table 2 shows the fiber composition for apparel, where cotton has the highest proportion among all other fibers consumed in Australia. Overall, cotton production is a high energy- and water-intensive process that causes several environmental impacts. Mechanical and chemical recycling of waste cotton textiles can reduce environmental impacts instead of sending all the cotton resources to the landfill. In both recycling processes, the volume of textile waste which is not recycled is disposed of in landfills. Moazzem et al. (2021b) have taken a similar approach, where a nonrecycled portion of waste textile is sent to a landfill.

**Table 2:** Composition of the textile fiber considered in the study

Fiber	Component	Amount (%)
Natural fiber and regenerated fiber (viscose)	Cotton	54.07
	Wool	1.57
	Viscose	3.41
	Flax	5.35
Synthetic fiber	Polyester	31.85
	Acrylic	3.46
	Nylon	0.29

### System boundary and functional unit

**Fig. 2:**
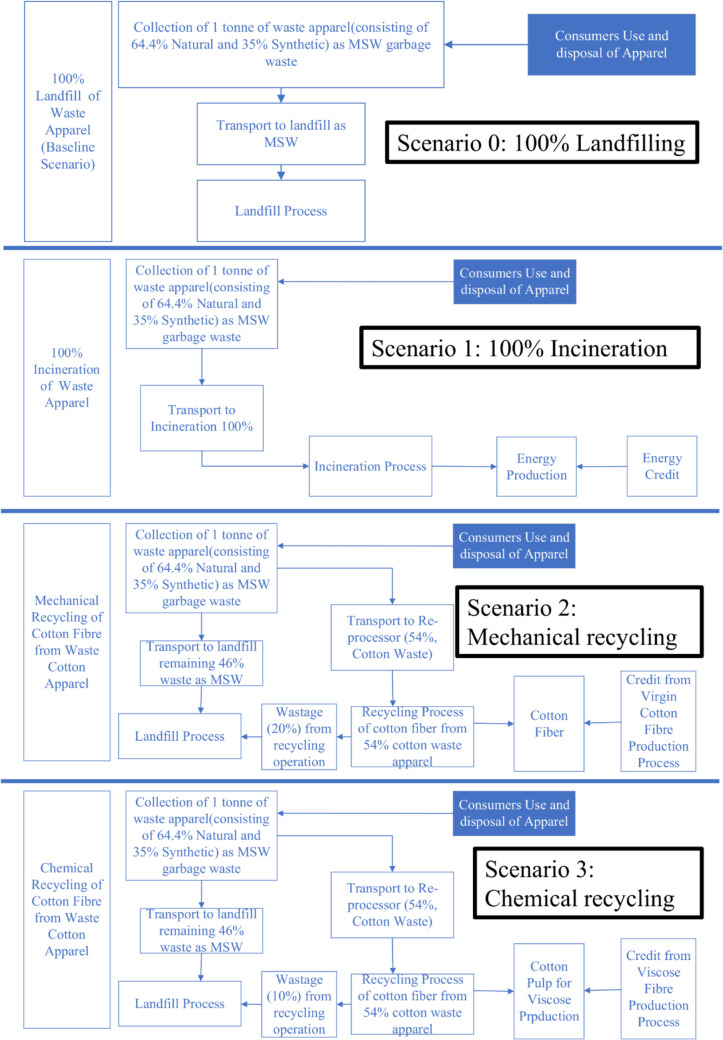
System boundary of waste textile (MSW, municipal solid waste)

Figure 2 shows that the system boundary of this study initiates from the collection of waste apparel to various EoL scenarios. Raw material production, apparel manufacturing, and consumer use stages are not included in this study, as the aim of this study is to assess the energy demand and carbon footprint of EoL treatment scenarios. The system boundary encompasses the scenarios, as mentioned in the goal and scope.

In scenario 0 (baseline scenario), discarded apparel is not subjected to any recycling and is directly disposed of in a landfill as the EoL treatment option. In 100% incineration (Scenario 1), all the discarded apparels are incinerated. Energy recovery via incineration was considered as energy credit. The recycling-related scenarios (Scenario 2: mechanical recycling and Scenario 3: chemical recycling) involve the processing of discarded apparel as EoL management alternatives.

The scope of this study is expanded, encompassing credits derived from preventing virgin production processes for similar products within the system boundary (Schmidt et al. 2016). The prevented processes vary from one scenario to another, depending on the secondary materials generated from the recycling process. This study's functional unit (f.u.) is 1000 kg (1 ton) of waste apparel in all scenarios.

#### Description of the scenarios

##### Scenario 0: 100% landfilling (baseline scenario)

Scenario 0 involves collecting discarded textiles from the kerbside waste stream using an MSW collection system. It examines the amount of emissions (i.e., in kg CO_2_eq) associated with the landfilling process only. Typically, the landfilling process involves methane gas collection; however, in this study, it was excluded.

The landfill operations require an energy input of 0.8 kWh per ton of textile waste, which is equivalent to 2.88 MJ of electricity and 38.6 MJ of diesel fuel (Moazzem et al. 2021b). Transporting the waste from kerbside to a designated landfill site would require a vehicle, and, in this study, a 21-ton municipal waste collection lorry was considered. Transport distance is also a critical parameter related to transport emission, and a standard transport distance of 20 km was assumed for this purpose. The transportation process, as catalogued in the Ecoinvent database, was utilized. This assumption is grounded in the simplified analysis of transportation for municipal waste collection, encompassing both landfill and recycling facilities, as outlined by Moazzem et al. (2021b). Table 3 shows the material and energy input and outputs for Scenario 0. The data from Table 2 was utilized to estimate the amount of various types of (waste) materials (i.e., summed as 1000 kg) as inputs for all the scenarios. The background data for the materials were collected from Ecoinvent and the CCaLC database embedded in the CCaLC2 software. In the database, CO_2_eq emissions and energy-related quantity are generally assigned on the basis of 1 kg. The word “UserDefned” shown in Table 3 refers to specific amount given as input for the scenario.

**Table 3:** Material and energy input and output for “Scenario 0” utilized in the model

	Amount	Unit	Database/reference
Material input as waste			
Acrylic fabric	34.6	kg	User-defned/materials/^a^
Cotton fiber	540	kg	CCaLC/materials/biofuels/biofeedstock^b^
Flax fiber	53.5	kg	User-defined/materials/^a^
Nylon 6, at plant	2.9	kg	Ecoinvent/materials/all materials^b^
Polyester fiber	319	kg	User-defined/materials/^a^
Viscose (Asia) fiber	34.1	kg	CCaLC/materials/all materials^b^
Wool, sheep, at farm, USA	15.7	kg	Ecoinvent/materials/all materials^b^
Energy input			
Diesel (burned)	38.6	MJ	CCaLC/energy^b^
Electricity, hard coal, at coal mine power plant	2.88	MJ	Ecoinvent/energy^b^
Transport input			
21-ton municipal waste collection lorry	20	km	Moazzem et al. (2021b)
Material output as waste	1000	kg	Calculated (generic)

^a^According to the fiber composition found in Australian textile waste, presented in Table 2 and converted for 1000 kg of textile waste

^b^The database included in the CCalC2 software. These applied to subsequent tables (e.g., Tables 4, 5, and 6)

##### Scenario 1: 100% incineration

Scenario 1 includes transporting apparel waste from the kerbside garbage stream to the landfill site, coupled with the operational undertakings conducted within the landfill confines of the incineration process. 

Moazzem et al. (2021b) mentioned that the incineration or energy recovery method could be more practical in Australia. However, Australian Fashion Council (2022) reported that Australia’s average apparel import amount is 373,000 tons each year, of which only 15,000 tons (e.g., 4.02% of the total apparel waste) go for energy recovery.

Similar to Scenario 0, a transportation distance of 20 km between the waste collection site and the incineration facility was considered for Scenario 1, using a 21-ton municipal waste collection lorry. It is estimated that incinerating one ton of textile waste requires 225 MJ of diesel and 1290 MJ municipal waste incinerator in Sweden (Bodin 2016). This data was considered for Australia as inputs for the model associated with Scenario 1. The municipal solid waste incinerator processing mentioned in the relevant Ecoinvent database in CCalC2 software was utilized in this case. Table 4 provides a detailed overview of the material and energy input and outputs for Scenario 1, demonstrating the thoroughness of our research and data analysis.

**Table 4:** Material and energy input and process outputs for “Scenario 1” utilized in the model

	Amount	Unit	Database/reference
Material input as waste			
Acrylic fabric	34.6	kg	User-defined/materials/
Cotton fiber	540	kg	CCaLC/materials/biofuels/biofeedstock
Flax fiber	53.5	kg	User-defined/materials/
Nylon 6, at plant	2.9	kg	Ecoinvent/materials/all materials
Polyester fiber	319	kg	User-defined/materials/
Viscose (Asia) fiber	34.1	kg	CCaLC/materials/all materials
Wool, sheep, at farm, USA	15.7	kg	Ecoinvent/materials/all materials
Energy input			
Diesel (burned)	225	MJ	CCaLC/energy
Treatment of municipal solid waste, incineration, CH	1290	MJ	Ecoinvent/energy
Transport input			
21-tonne municipal waste collection lorry	20	km	Moazzem et al. (2021b)
Material/energy output			
Energy production	1920	kg	Calculated
Heat production	1080	kg	Calculated

##### Scenario 2: mechanical recycling of waste cotton fraction

In mechanical recycling, discarded cotton waste undergoes a sequence of operations. It is initially broken down into small pieces through a machine, followed by shredding, blending, and combing. Eventually, these steps lead to the transformation of the waste into recycled cotton fiber. It is estimated that within 1000 kg of textile waste, approximately 540 kg are composed of cotton (Moazzem et al. 2021b). The non-recycled textile waste was allocated to landfill disposal in the mechanical recycling process.

On top of that, in the mechanical recycling process, approximately 108 kg (i.e., 20% of 540 kg cotton waste) of textile waste is considered a loss. The total cotton fraction of the textile waste for further processing is approximately 432 kg (of recycled cotton). The quality of recycled cotton via the mechanical process is a critical issue, as reported by Schmidt et al. (2016) and Zamani et al. (2015), for which the authors suggested including virgin cotton fiber (as a blending) to enhance the quality of the recycled cotton as output from the process. This blending process involves a mix of 55% recycled cotton with 45% virgin cotton, aiming to elevate the quality of the final product, as prescribed by Moazzem et al. (2021b) and Schmidt et al. (2016). This scenario utilized such a concept to effectively enhance the quality of the recycled cotton, displacing an equivalent amount of virgin cotton on a one-to-one basis. The Ecoinvent process associated with cotton fiber production was employed in the model to assess the avoided production of virgin cotton.

The mechanical recycling of cotton utilizes electricity generated from hard coal, as per coal mine power plant standards, with an energy consumption of 194 MJ for the processing of the cotton fraction. Such approximation was made because majority of the grid connected power generation in Australia comes from coal power plants. Table 5 lists all the energy inputs including landfill process, virgin cotton production, and mechanical processing, as well as other material inputs and outputs.

**Table 5:** Material and energy input and process outputs for “Scenario 2” utilized in the model

	Amount	Unit	Database/reference
Material input as waste			
Acrylic fabric	34.6	kg	User-defned/materials/
Cotton fiber	540	kg	CCaLC/materials/biofueIs/biofeedstock
Flax fiber	53.5	kg	User-defined/materials/
Nylon 6, at plant	2.9	kg	Ecoinvent/materials/all materials
Polyester fiber	319	kg	User-defined/materials/
Viscose (Asia) fiber	34.1	kg	CCaLC/materials/all materials
Wool, sheep, at farm, USA	15.7		Ecoinvent/materials/all materials
Material input as quality improvement			
Cotton production, USA	354	kg	Ecoinvent/materials/agriculture
Energy input			
Diesel (burned)	17.8	MJ	CCaLC/energy
Electricity, hard coal, at coal mine power plant	205	MJ	Ecoinvent/energy
Electricity, hard coal, at coal mine power plant	194	MJ	Ecoinvent/energy
Electricity, hard coal, at coal mine power plant	1.32	MJ	Ecoinvent/energy
Transport input			
21-ton municipal waste collection lorry	20	km	Moazzem et al. (2021b)
Material/energy output			
Waste to landfill after mechanical recycling	108	kg	CCaLC/waste
Direct landfill (without cotton)	460	kg	CCaLC/waste
Recycled cotton	785	kg	Calculated

##### Scenario 3: chemical recycling of waste cotton fraction

The chemical recycling process of cotton fiber considers the 540 kg cotton component out of a total of 1000 kg of textile waste. Similar to mechanical recycling, the remaining portion of textile waste is allocated to landfill disposal. This specific recycling process commences with a pre-treatment involving a sodium hydroxide (50%) solution and then sulfuric acid (96%) for acid hydrolysis. The input for the chemical recycling process is detailed as 540 kg of cotton garments that have been sorted, shredded, and washed.

Following this initial stage, the process requires several cleaning steps to thoroughly remove acid, with an estimated water usage of about 23,760 L treating the cotton fraction. Both electricity and thermal energy were given as input in the system for the recycling operation. Schmidt et al. (2016) utilized such an approach and considered biomass-based energy sources for thermal energy used in the process. In the case of Australia, biomass-based thermal energy sources for processing waste have yet to be operational. Thus, in this study, “natural gas, burned in a gas turbine, global” was given as input for a thermal energy source. This choice reflects the prevalent use of natural gas in the thermal production systems of conventional manufacturing processes, providing a realistic basis for the analysis. In producing virgin pulp, a dissolving process necessitates 0.416 kWh of electricity per kilogram, as per Schmidt et al. (2016). Table 6 shows the material and energy input and outputs for Scenario 3.

**Table 6:** Material and energy input and process outputs for “Scenario 3” utilized in the model

	Amount	Unit	Database/reference
Material input as waste			
Acrylic fabric	34.6	kg	User-defned/materials/
Cotton fiber	540	kg	CCaLC/materials/biofuels/biofeedstock
Flax fiber	53.5	kg	User-defined/materials/
Nylon 6, at plant	2.9	kg	Ecoinvent/materials/all materials
Polyester fiber	319	kg	User-defined/materials/
Viscose (Asia) fiber	34.1	kg	CCaLC/materials/all materials
Wool, sheep, at farm, USA	15.7	kg	Ecoinvent/materials/all materials
Chemical inputs for processing			
Sodium hydroxide, 50% in H_2_O, production mix, at plant	4.2	kg	Ecoinvent/materials/chemicals
Sulfuric acid from viscose production, at plant	108	kg	Ecoinvent/materials/chemicals
Tap water, at user, Europe	23,800	kg	Ecoinvent/materials/water
Energy input			
Diesel (burned)	17.8	MJ	CCaLC/energy
Electricity, hard coal, at coal mine power plant	205	MJ	Ecoinvent/energy
Electricity, hard coal, at coal mine power plant	225	MJ	Ecoinvent/energy
Electricity, hard coal, at coal mine power plant	1.32	MJ	Ecoinvent/energy
Natural gas, burned in gas turbine, global	3,634	MJ	Ecoinvent/energy
Transport input			
21-ton municipal waste collection lorry	20	km	Moazzem et al. (2021b)
Material/energy output			
Waste to landfill after chemical recycling	108	kg	CCaLC/waste
Direct landfill (without cotton)	460	kg	CcaLC/waste
Wastewater	23,800	kg	
Cotton pulp for viscose production	486	kg	Calculated

#### CCalC2 software and data collection

To analyze the scenario quantitatively, the CCalC2 and the Ecoinvent database embedded in the software were utilized to estimate the scenarios' CO_2_eq and energy demand. In addition, as seen from the descriptions of the individual scenarios, various literature data were also utilized with citations.

The CCalC2 software was developed by the University of Manchester following internationally accepted standards such as ISO 14044 and PAS2050 (University of Manchester 2016). Amin et al. (2021) have effectively utilized this software for various cotton knitwear production processes in Bangladesh. Thapa et al. (2022) utilized the software for food waste treatment, while Güller and Balcı (2018) used the software to assess carbon footprint of waste water treatment plant.

Using secondary data in CF analysis is a common practice. For example, Millward-Hopkins et al. (2023) used data from a review study authored by Munasinghe et al. (2021) on lifecycle inventory, for the case of UK clothing economy. In another study, Amicarelli and Bux (2022) used the emission and energy-related data from Youhanan (2013) and Bodin (2016).

## Results and discussion

### Energy demand analysis

**Fig. 3:**
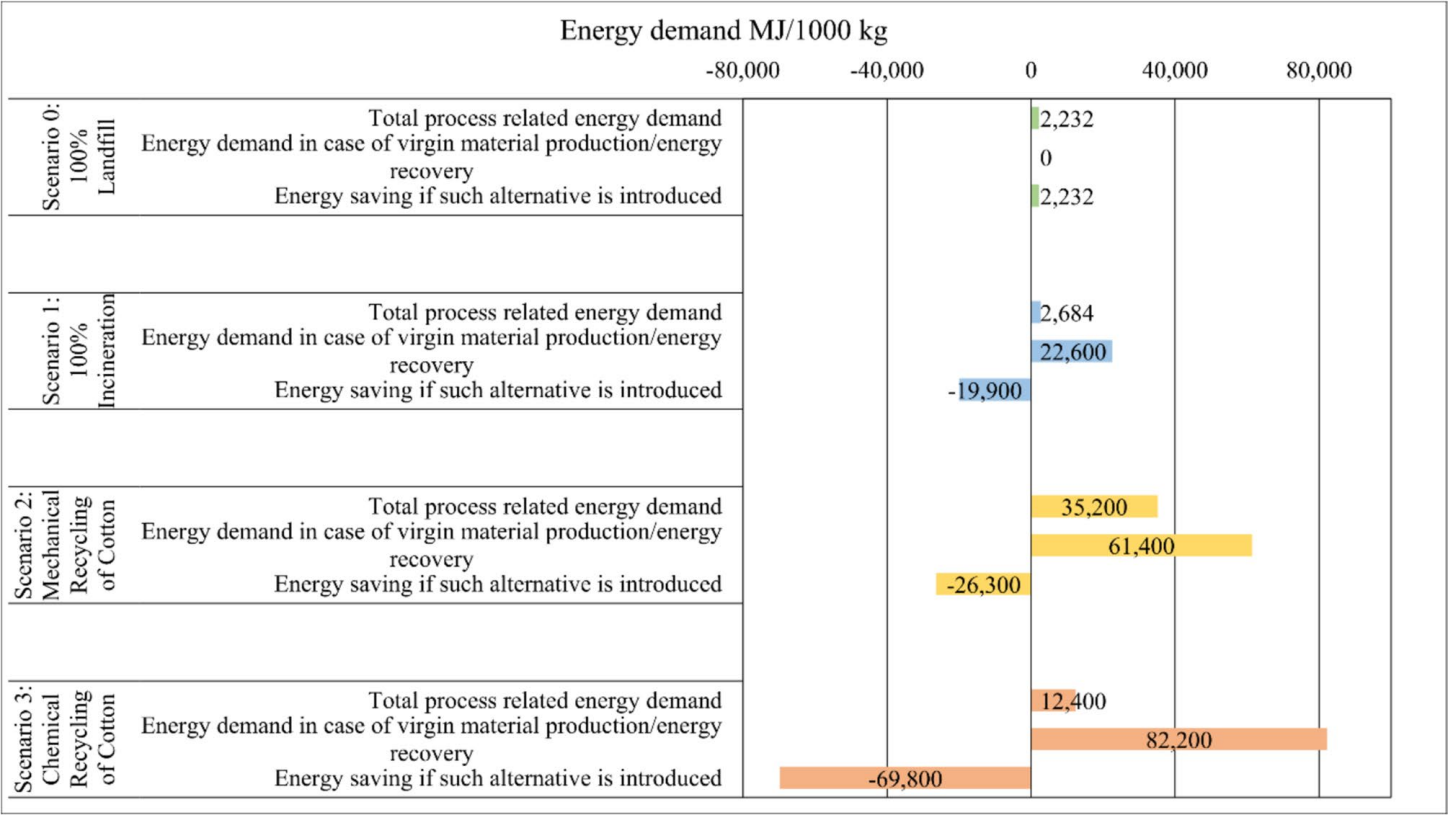
Comparative energy demand per functional unit of various EoL scenarios of textile waste

Figure 3 shows energy demand for 1000 kg of textile waste under various EoL management strategies, which are Scenario 0, 100% landfilling (baseline scenario); Scenario 1, 100% incineration; Scenario 2, mechanical recycling of cotton; and Scenario 3, chemical recycling of cotton. The energy demand trend for these scenarios can be interpreted in three distinct ways. Firstly, total process-related energy demand; secondly, energy demand in case of virgin material/energy production (business as usual); and thirdly, energy saving if such alternative is chosen (e.g., mechanical recycling/chemical recycling/incineration). These results are the additional CF analyses from the CCalC2 software.

In Scenario 0, the energy demand of virgin material/energy production was defined as zero (“0”), as all waste textile goes to the landfill. In this study, we did not consider a landfill gas recovery process. This is why the total process-related energy demand and energy saving amount represent the same (2232 MJ/f.u.). However, for Scenario 1, 19,900 MJ/f.u. was estimated as an energy saving, as the process generated electricity and thermal energy as the co-product. The total process-related energy demand for 100% incineration was found to be 22,600 MJ/f.u. Thus, incineration offers certain benefits of energy recovery, while no recovery has been considered for the landfill process. In some studies, such as Moazzem et al. (2021b), methane collection was considered as a process of energy credit which might create insignificant energy recovery compare to incineration. Zamani et al. (2015) indicated that incineration could generate 12,400 MJ/ton of alternative electricity, suggesting a potential energy recovery aspect in the incineration process.

Notably, both mechanical and chemical recycling illustrated negative energy demands which are around − 26,300 MJ/f.u. and − 69,800 MJ/f.u., respectively, implying energy credit. This energy credit is a direct result of material recovery. In the case of mechanical recycling, 61,400 MJ/f.u. energy is required to grow virgin cotton (i.e., 432 kg). As mentioned earlier, the recycled cotton will replace virgin cotton in the same proportion (1:1), such approximation has been considered in case of energy demand trend of the recycling process. The energy credit (i.e., − 26,300 MJ/f.u.) corresponds to producing the same amount of cotton as recovered in the process. On the other hand, in the chemical recycling process, the overall energy demand was found to be 12,400 MJ/f.u. When virgin viscous pulp could be replaced by recycled viscous pulp (i.e., 486 kg of dry dissolving pulp), the estimated energy demand for such virgin product would be 82,200 MJ/f.u. In the chemical recycling process, a total of 69,800 MJ/f.u. could be saved by considering a replacement of virgin pulp with the recycled viscous pulp. In both Scenarios 2 and 3, the energy saving corresponds to the material recovery process, while in incineration the energy credit is based on energy recovery from the process (i.e., thermal, and electrical energy). Muthu et al. (2012) utilized SimaPro to demonstrate that recycling process-waste significantly reduces energy demand, while Zamani et al. (2015) highlighted that integrating multiple recycling techniques could save up to 169 GJ of primary energy per ton of textile waste.

### Carbon footprint analysis

**Fig. 4:**
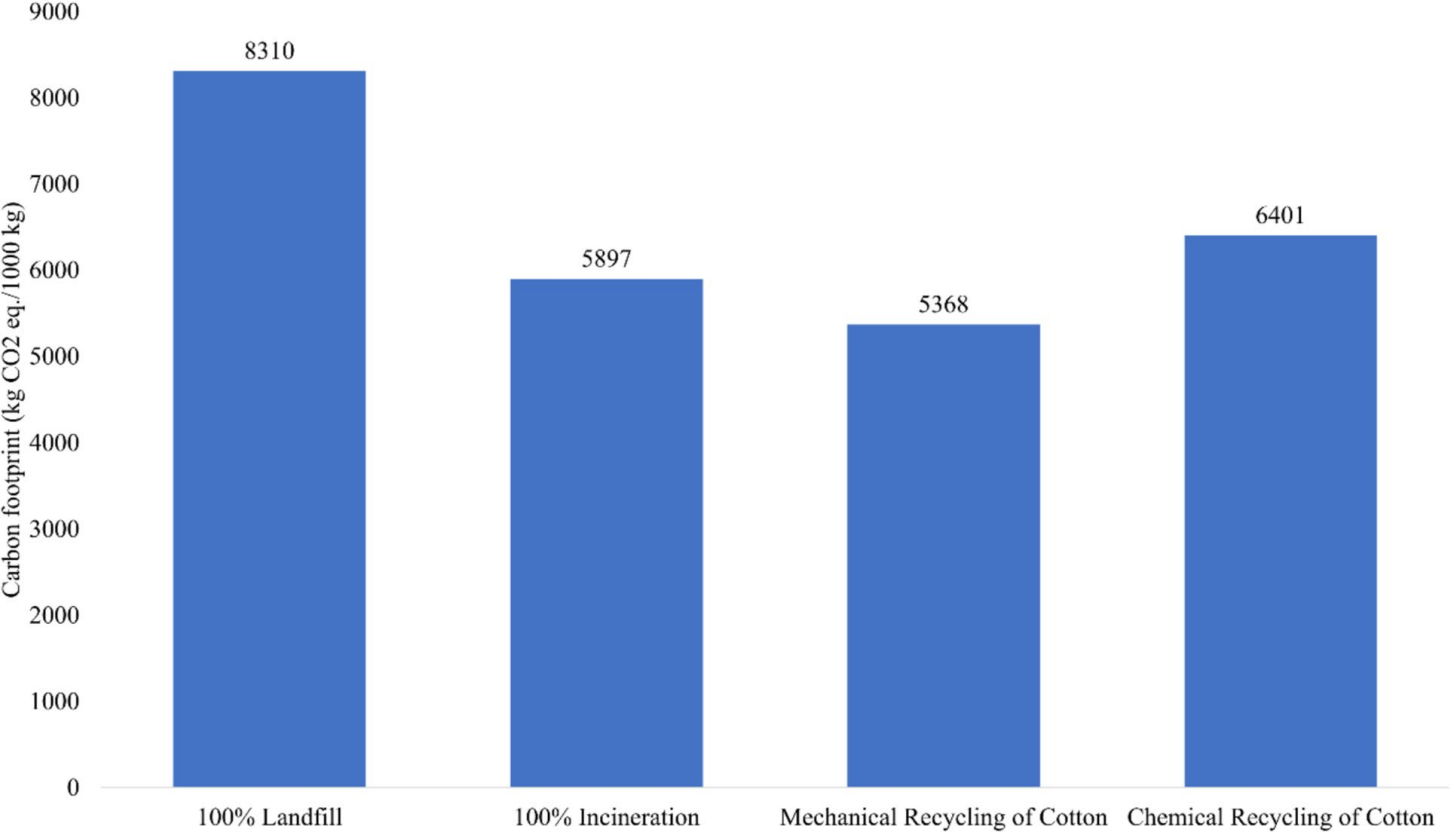
Comparative CF of various EoL scenarios of textile waste

Figure 4 represents the CF of 1000 kg textile waste where 100% landfill contributes the most, while mechanical recycling is the most minor contributor. The environmental impact assessment of the 100% landfill scenario, as quantified by the model from this study, reveals that 1000 kg of textile waste generates approximately 8310 kg of CO_2_eq, which is by far the worst practice in CO_2_ emissions. The CF varies substantially in the modelling process, and comparisons between the processes can often be made. Nevertheless, energy sources, energy consumption in various processes, and the diversity of materials under consideration provide different output results. Using the ReCipe method in Open LCA software, Moazzem et al. (2021b) estimated emissions under the climate change potential (CCP) would be 218 kg CO_2_eq/ton of natural apparel and 34.8 kg CO_2_eq/ton of synthetic apparel. It is to be noted that energy production from methane gas generation at the landfill was considered in their modelling.

In exploring waste management alternatives within the Australian context, where incineration is not a prevalent practice, a hypothetical model was constructed to examine the incineration of a functional unit. Kwinana Waste to Energy Project suggests around 36 MW of baseload power could be generated annually from an estimated 400,000 tons of commercial and industrial refuse, pre-sorted construction and demolition debris, and municipal solid waste in Western Australia (Australian Renewable Energy Agency (ARENA) 2023). Scenario 1 would emit 5,897 kg CO_2_eq according to the model, thereby critically assessing the environmental impact of adopting incineration as a textile waste management strategy in Australia. In Fig. 4, mechanical recycling of cotton fiber is identified as the process with the lowest CO_2_eq emissions (i.e., 5368 kg CO_2_eq). In the framework of this environmental model, recycled cotton is treated as a co-product. This approach involves subtracting the CF of the co-product from the overall CF of the system, thus aligning with the LCA principles and emphasizing the importance of reducing environmental impact through efficient resource utilization and waste management strategies. The environmental impact of the chemical recycling process is quantified in terms of its CF and calculated to be 6401 kg of CO_2_eq. This metric is crucial for evaluating the sustainability and efficacy of the chemical recycling method in the broader framework of textile waste management and environmental conservation strategies.

In terms of material recovery and energy saving, chemical recycling is the best solution. However, for CO_2_eq emissions, the recycling process showed the second largest carbon-intensive process, which pointed out the critical technology selection. The viscous pulp recovery has added advantages, considering the water requirement of chemical recycling as compared to growing cotton. On the other hand, mechanical recycling is a less carbon-intensive process. However, energy saving from the process was almost half of that for chemical recycling. The loss of fiber associated with the mechanical recycling process (i.e., 20% material wastage during the recycling process) could be the probable reason for such less energy-saving potential. Energy saving in the mechanical recycling and incineration processes showed a similar pattern, following the CO_2_ emission trend. These findings are consistent with Muthu et al. (2012), who demonstrated that recycling process waste reduces CF compared to conventional disposal, emphasizing the need for ecological design to enhance recyclability and minimize emissions at the design stage. Thus, the priority of EoL management is to reduce the process-level CO_2_ emissions, followed by mechanical recycling as the best alternative.

Chemical recycling provides greater flexibility in recovering recycled cotton, indirectly minimizing the production process-level energy requirements. In other words, the environmental and economic benefits should be seen from the supply chain perspective. One suggestion could be implementing an emission trading scheme for the chemical recycling process like other industrial operations, which might create a win–win scenario and acceptable solution to reduce emissions from the operation compared to mechanical recycling and incineration. If the loss of fiber around mechanical recycling could be minimized and more energy-efficient mechanical processes could be implemented along with carbon offset schemes, mechanical recycling could be the best EoL management approach among all the options. Zamani et al. (2015) conducted an LCA in Sweden and revealed that textile recycling through material reuse, emissions can be reduced approximately 8 tons of CO₂eq per ton of waste, indicating the impact of recycling methods and energy recovery on carbon footprint reduction. Landfilling should be eliminated by any means as this is a process of losing material resources, involving high emissions and no benefits to the natural environment.

The shift from traditional landfilling to alternative waste management strategies holds immense potential, offering significant environmental benefits. This transition can notably reduce global warming potential and energy consumption, in addition to water savings. Furthermore, the adoption of textile recycling technologies can play a pivotal role in significantly lowering the overall impact of climate change, inspiring a more sustainable future.

Based on the study, it is seen that there is a clear requirement of adopting recycling-focused technologies to avoid potential CO_2_ emissions derived from landfilling, which is currently widely practiced in Australia. From this research, policymakers and local waste management authorities will understand the benefits of adopting mechanical and chemical recycling for which substantial investment is required. Extended producer responsibility could be the mechanism making producers and importers responsible for managing and investing in recycling processes. Furthermore, to make such analysis presented in this study usable at the national level, there should be development around a database on waste generation, processing, and recycling infrastructure, and technology use. Consumer behavior around the disposal practice of textile waste with household garbage could be minimized by raising awareness and providing incentives.

## Conclusion

This study showed a substantial opportunity to deploy alternatives rather than using landfills as the final destination of textile waste in Australia by conducting energy demand and carbon footprint analysis. The mechanical recycling process is estimated to emit around 5368 kg CO_2_eq/1000 kg — the least CO_2_ emitting process. Incineration showed 5897 kg CO_2_eq/1000 kg CO_2_ emissions, similar to mechanical recycling. The incineration option could only be pursued if (thermal) energy recovery and electricity production are considered. Within the framework of this study, the outputs from each disposal method are treated as co-products, thereby offsetting the environmental impact and explaining the comparative benefits of each method.

This study provides a preliminary quantitative assessment of the carbon emissions and energy demand from each scenario with limitations, primarily around data and relevant processes. The study focuses on cotton textile waste, utilizing secondary data sources (i.e., literature and predefined databases in CCalC2). The use of secondary data is a common practice in carbon footprint exercise. This study acknowledges the need for primary data to develop a more robust model and calls for future research to address potential trade-offs, such as the CO_2_ emissions from mechanical and chemical recycling and waste-to-energy incineration. Methane gas recovery in the landfill scenario has yet to be considered. The incineration process is not typical for Australian textile waste management, so this study utilized the process from the Ecoinvent database. More lab-based studies at the initial stage are required to quantify the process's CO_2_ emissions and energy demand.

There is a continuous effort to make the mechanical and chemical processes effective and efficient globally. Thus, there will be a change in technology as the energy requirements and emission patterns change. This study undertook a preliminary exercise of modelling the mechanical and chemical recycling process, which could be improved considering more contextual factors and onshore recycling processes. Researchers working in technology development for textile waste processing should also consider CO_2_ emissions and energy demand-related estimations so that more Australia-specific carbon footprint reduction exercises could be done. The Australian national-level target has been set for a 50% reuse rate by 2030, but no clear indication has been provided regarding recycling. The study emphasizes the importance of incorporating other environmental aspects like toxicity and water usage in future calculations and highlights the need for investment in Australia's recycling sector.

Future research should focus on improving recycling technologies as well as consider synthetic waste, optimizing energy efficiency, including usage of renewable energy, and developing comprehensive policy frameworks to support sustainable textile waste management. Furthermore, future research should also conduct a sensitivity analysis in the carbon footprint-related study, revealing the relationship between recycling rate and CO_2_ emissions. Such sensitivity analysis should also consider the potential CO_2_ emissions when 1 ton of cotton textile is recycled (without adding virgin cotton for quality improvement) via mechanical and chemical recycling. National-level recycling targets could also be included in such sensitivity analysis for Australia (i.e., in the EU, the textile waste recycling target is 55% by 2025, 60% by 2030, and 65% by 2035). The shift from landfilling to recycling and energy recovery is imperative for achieving long-term environmental and economic benefits, supporting Australia's commitment to sustainability and climate change mitigation.

## Data Availability

The data supporting the findings of this study are available within the paper.
